# Meningitis and Bacteremia Caused by *Streptococcus suis*: First Case Report in Colombia

**DOI:** 10.1155/crdi/4842782

**Published:** 2026-04-01

**Authors:** Sanchez P. Santiago, Corredor N. Luis, Peña T. Carlos, Botero Z. Victor, Rodriguez N. Antonio

**Affiliations:** ^1^ Faculty of Medicine, Unisanitas, Bogotá, Colombia

**Keywords:** bacteremia, bacterial meningitis, Colombia, emerging pathogen, MALDI-TOF, *Streptococcus suis*, zoonotic infection

## Abstract

The first documented instance of acute bacterial meningitis caused by *Streptococcus suis* in Colombia has been reported. A 69‐year‐old male resident of a rural area with a history of direct contact with pigs presented to the clinic with a persistent fever, severe headaches, and altered consciousness. Cerebrospinal fluid analysis showed neutrophilic pleocytosis, increased cerebrospinal fluid protein levels, and decreased glucose due to consumption. Initial culture and confirmation by mass spectrometry MALDI‐TOF identified *S. suis*, with an MLSB resistance profile and resistance to tetracyclines, but with sensitivity to beta‐lactams, quinolones, and vancomycin. The patient received empirical treatment with ceftriaxone, vancomycin, and ampicillin, with favorable progress from the first 24 h, completing 10 days of guided therapy, restricted to ceftriaxone given the susceptibility profile on the antibiogram and its bioavailability in the central nervous system without immediate sequelae. This finding represents direct evidence of the presence of *S. suis* in humans in the country, raising the possibility of underdiagnosis associated with a lack of clinical suspicion and limitations in the microbiological capacity of local laboratories. The absence of classic meningeal signs in the clinical presentation underscores the necessity for a high index of suspicion, particularly in rural areas with exposure to pigs. Furthermore, the importance of considering this emerging zoonosis in the differential diagnosis of bacterial meningitis is reinforced, given its potential to cause permanent neurosensory complications. The case study outlined in this text emphasizes the pressing need to reinforce epidemiological surveillance, expand diagnostic capacity, and enhance health personnel’s awareness of this zoonotic pathogen in the region. These measures are crucial for enhancing early detection and optimizing treatment outcomes.

## 1. Introduction

*Streptococcus suis (S. suis)* is an emerging zoonotic Gram‐positive coccus that colonizes pigs and can cause invasive infections in humans, including meningitis, sepsis, endocarditis, and arthritis. The transmission of the disease occurs primarily through contact with infected animals or undercooked meat, and the affected population is predominantly composed of rural workers and individuals with occupational exposure [1, 2]. Since its initial documentation in humans in 1968, the prevalence of the condition has increased, predominantly not only in Asia but also in South America, where 47 cases have been reported between 1995 and 2024, 85% of which were classified as meningitis, and up to 60% of which resulted in hearing loss as a frequent sequela, in addition to ataxia [2–4]. Most cases are associated with Serotype 2, which expresses virulence factors such as *sly, epf,* and *mrp* [3]. Argentina accounts for most cases within the South American region. In contrast, Colombia, despite having an even larger pig industry, has not reported any human infections. This suggests underreporting due to clinical ignorance, diagnostic limitations, or the circulation of less virulent strains [3, 4]. The objective of this article is to report the first case of this infection in Colombia.

## 2. Case Presentation

A 69‐year‐old male farmer from a rural area of Antioquia, Colombia (2550 m above sea level), with occupational exposure to cattle and pigs and no known past medical history, presented to a healthcare facility in Yarumal, Antioquia, on February 18, 2025, with an eight‐day history of fever (38.5°C–39.0°C), malaise, and severe holocranial headache that awakened him at night. During the 48 h prior to presentation, relatives reported paroxysmal episodes described as transient “disconnection” associated with delirium.

On admission, the patient appeared ill, drowsy but arousable, and disoriented in time and place. Vital signs showed temperature 39°C, heart rate 120 beats/min, blood pressure 124/74 mmHg, and dry mucous membranes. No meningeal signs or focal neurological deficits were observed. Lumbar puncture was performed, and cerebrospinal fluid (CSF) culture yielded *S. suis* (Table 1).

**TABLE 1:** Antibiogram of *S. suis* cerebrospinal fluid culture: *S. suis* 95% probability, VITEK.

Antimicrobial agent	MIC (μg/mL)	Interpretation
Ampicillin	≤ 0.25	Susceptible
Cefotaxime	≤ 0.12	Susceptible
Ceftriaxone	≤ 0.12	Susceptible
Levofloxacin	≤ 0.25	Susceptible
Moxifloxacin	0.12	Susceptible
Erythromycin	≥ 8	Resistant
Clindamycin	≥ 1	Resistant
Linezolid	≤ 2	Susceptible
Vancomycin	≤ 0.12	Susceptible
Tetracycline	≥ 16	Resistant
Chloramphenicol	4	Susceptible

*Note:* Identification probability of 95% by the VITEK system. Antimicrobial susceptibility interpreted according to CLSI breakpoints for viridans group streptococci due to the absence of species‐specific criteria for *S. suis*.

Contrast‐enhanced cranial computed tomography demonstrated nonspecific supratentorial leukoencephalopathy without meningeal enhancement, collections, or abscesses. CSF cytochemical findings are summarized in Table 2. Blood cultures initially identified *Streptococcus thoraltensis* with a 95% probability using the VITEK system. The isolate was therefore referred to a national reference laboratory for confirmatory identification by matrix‐assisted laser desorption ionization–time of flight mass spectrometry (MALDI‐TOF), which confirmed *S. suis*. Immunodeficiency was excluded by a negative HIV test and normal immunoglobulin levels (IgG, IgA, and IgM). Transthoracic echocardiography was normal. The patient was treated with ceftriaxone for 10 days, and follow‐up blood cultures at 72 h were negative.

**TABLE 2:** Cerebrospinal fluid cytochemistry and serum immunoglobulin profile.

Color	White
pH	7
Erythrocytes (cel/mm^3^)	132
Leukocytes (cel/mm^3^)	1344 (Polymorphonuclear cells 90%, mononuclear cells 10%)
Proteins (mg/dL)	156.4
Glucose (mg/dL)	CSF glucose: 46 Serum glucose: 160 CSF/serum ratio: 0.25
IgG (mg/dL)	782.0
IgM mg/dL	64.0
IgA mg/dL	336.0

## 3. Discussion

This case represents the first microbiologically confirmed isolation of *S. suis* in CSF and blood from a Colombian patient with occupational pig exposure and clinical features consistent with bacterial meningitis. The presentation was subacute, characterized by severe headache, altered consciousness, and absence of classic meningeal signs, a pattern previously described in immunocompetent older adults [1, 2, 5].

CSF analysis demonstrated findings typical of bacterial meningitis, including neutrophilic pleocytosis (1344 cells/mm^3^; 90%), elevated CSF protein (156.4 mg/dL), and hypoglycorrhachia (46 mg/dL) with a corresponding serum glucose of 160 mg/dL (ratio 0.25). Opening pressure was within normal limits. These findings strongly supported the diagnosis. The blood culture isolate was initially identified as *S. thoraltensis* with a 95% probability by the VITEK automated system; however, confirmatory MALDI‐TOF analysis identified the organism as *S. suis*. Misidentification of *S. suis* by automated phenotypic systems such as VITEK has been well documented due to overlapping biochemical profiles with other streptococcal species, highlighting the importance of confirmatory identification methods to ensure accurate species‐level diagnosis.

Antimicrobial susceptibility testing demonstrated sensitivity to ampicillin, ceftriaxone, cefotaxime, vancomycin, linezolid, moxifloxacin, and levofloxacin, allowing de‐escalation to ceftriaxone monotherapy as definitive therapy. Resistance to macrolides and lincosamides (erythromycin ≥ 8 μg/mL), tetracyclines (≥ 16 μg/mL), and clindamycin (≥ 1 μg/mL) was observed, consistent with the MLSB phenotype frequently reported in South American strains [2, 3]. These findings suggest that infection may have been present in Colombia but underrecognized. Rather than indicating true absence, the low number of reported cases likely reflects limited diagnostic capacity and low clinical suspicion in certain regions. This case, therefore, supports the probable circulation of potentially invasive strains within the country [3, 4].

A systematic review by Huong et al. found that up to 68% of *S. suis* infections present as meningitis, with neurosensory sequelae such as hearing loss occurring in 39.1% and vestibular dysfunction in 22.7%, along with a mortality rate of 12.8% [5]. None of these complications was observed in this patient. This report highlights that *S. suis* should be considered in the differential diagnosis of bacterial meningitis, particularly in rural populations with occupational exposure to animals. Although this is a single case, its microbiological confirmation, clinical course, and response to therapy are consistent with previous reports, suggesting applicability to similar epidemiological settings. Strengthening laboratory diagnostic capacity and surveillance for emerging zoonotic pathogens is therefore essential.

Specific susceptibility breakpoints for *S. suis* are lacking, and interpretive criteria for the viridans group streptococci are commonly applied. In general, this organism remains susceptible to beta‐lactams such as penicillin and third‐generation cephalosporins, although resistant isolates have been reported, particularly to penicillin, macrolides, and tetracyclines. Penicillin G remains the preferred first‐line therapy, while ceftriaxone, ampicillin, and chloramphenicol have also demonstrated efficacy. Corticosteroids such as dexamethasone have been investigated for the prevention of neurological sequelae, but current evidence does not support their routine use [6].

In a similar case reported by Dantas et al., an elderly patient with pork exposure received initial therapy with ceftriaxone, ampicillin, and dexamethasone followed by de‐escalation to ceftriaxone monotherapy for 14 days with a favorable outcome. In contrast, the present patient achieved clinical resolution with a 10‐day regimen, which was supported by rapid clinical improvement within 24 h, negative follow‐up cultures, absence of neurological complications, and sustained clinical stability throughout hospitalization [2].

Finally, MALDI‐TOF played a pivotal role in confirming the etiologic agent. MALDI‐TOF mass spectrometry has emerged as a rapid and reliable method for bacterial species identification and has significantly improved diagnostic accuracy compared to conventional phenotypic methods. These findings reinforce the value of MALDI‐TOF as a rapid, reliable, and cost‐effective diagnostic tool, particularly when discrepancies arise between automated identification systems and clinical suspicion.

## 4. Conclusion

This case confirms the presence of *S. suis* as a human pathogen in Colombia and highlights the importance of considering this zoonotic agent in patients presenting with bacterial meningitis and occupational exposure to pigs. The report underscores the risk of underdiagnosis in regions with limited microbiological resources and emphasizes the value of advanced diagnostic techniques such as MALDI‐TOF for accurate identification. Strengthening clinical awareness, laboratory capacity, and zoonotic surveillance is essential to improve early recognition and optimize outcomes in similar settings.

## Funding

The authors declare that they have no sources of funding for the preparation of the manuscript.

## Ethics Statement

Written informed consent for publication was obtained from the patient.

## Conflicts of Interest

The authors declare no conflicts of interest.

## Data Availability

All data supporting the findings of this study are included within the article. Additional information is available from the corresponding author upon reasonable request.

## References

[1] Goyette-Desjardins G. , Auger J. P. , Xu J. , Segura M. , and Gottschalk M. , *Streptococcus suis*: An Emerging Human Pathogen, Clinical Infectious Diseases. (2014) 58, no. 5, 664–674, 10.1093/cid/cit798, 2-s2.0-84894283616.

[2] Ramos J. N. , da Silva Oliveira L. , Souza G. et al., Primer Caso Humano de Meningitis por *Streptococcus suis* en Brasil: Un Patógeno Zoonótico Emergente en América del sur, Revista da Sociedade Brasileira de Medicina Tropical. (2024) 57, e0012–e2024, 10.1590/0037-8682-0012-2024.

[3] Bakpatina-Batako J. , Ortiz-Almeida C. , Montealegre O. et al., *Streptococcus suis* Meningitis in South America: An Emerging Zoonosis, Emerging Infectious Diseases. (2025) 31, no. 1, 24–32.

[4] Koch E. , Fuentes G. , Carvajal R R. et al., Meningitis Bacteriana Aguda Por Streptococcus Suis en Criadores de Cerdos: Comunicación de los PRIMERos dos Casos En Chile, Revista Chilena de Infectología. (2013) 30, no. 5, 557–561, 10.4067/S0716-10182013000500015, 2-s2.0-84887275456. 24248173

[5] Huong V. T. , Ha N. , Huy N. T. et al., Epidemiology, Clinical Manifestations, and Outcomes of *Streptococcus suis* Infection in Humans, Emerging Infectious Diseases. (2014) 20, no. 7, 1105–1114, 10.3201/eid2007.131594, 2-s2.0-84902771911. 24959701 PMC4073838

[6] Huerta-Santana R. , Domínguez-Pinilla N. , López-Calleja A. I. et al., Meningitis Por *Streptococcus suis*: ¿Una Zoonosis Emergente?, REV Clinical. (2018) 11, no. 2, 82–89, 10.1016/j.labcli.2017.06.002, 2-s2.0-85023630725.

